# Sequence and gene content of a large fragment of a lizard sex chromosome and evaluation of candidate sex differentiating gene R-spondin 1

**DOI:** 10.1186/1471-2164-14-899

**Published:** 2013-12-17

**Authors:** Tariq Ezaz, Bhumika Azad, Denis O’Meally, Matthew J Young, Kazumi Matsubara, Melanie J Edwards, Xiuwen Zhang, Clare E Holleley, Janine E Deakin, Jennifer A Marshall Graves, Arthur Georges, Scott V Edwards, Stephen D Sarre

**Affiliations:** 1 Institute for Applied Ecology, University of Canberra, Canberra 2601, Australia; 2 La Trobe Institute of Molecular Biology, La Trobe University, Melbourne, VIC 3086, Australia; 3 Department of Organismic and Evolutionary Biology, Harvard University, 26 Oxford Street, Cambridge, MA 02138, USA

**Keywords:** ZW sex chromosomes, Genotypic sex determination (GSD), Temperature dependent sex determination (TSD), RSPO1, Squamata, Reptilia

## Abstract

**Background:**

Scant genomic information from non-avian reptile sex chromosomes is available, and for only a few lizards, several snakes and one turtle species, and it represents only a small fraction of the total sex chromosome sequences in these species.

**Results:**

We report a 352 kb of contiguous sequence from the sex chromosome of a squamate reptile, *Pogona vitticeps,* with a ZZ/ZW sex microchromosome system. This contig contains five protein coding genes (*oprd1, rcc1, znf91, znf131*, *znf180*), and major families of repetitive sequences with a high number of copies of LTR and non-LTR retrotransposons, including the CR1 and Bov-B LINEs. The two genes, *oprd1* and *rcc1* are part of a homologous syntenic block, which is conserved among amniotes. While *oprd1* and *rcc1* have no known function in sex determination or differentiation in amniotes, this homologous syntenic block in mammals and chicken also contains *R-spondin 1* (*rspo1*), the ovarian differentiating gene in mammals. In order to explore the probability that *rspo1* is sex determining in dragon lizards, genomic BAC and cDNA clones were mapped using fluorescence *in situ* hybridisation. Their location on an autosomal microchromosome pair, not on the ZW sex microchromosomes, eliminates *rspo1* as a candidate sex determining gene in *P. vitticeps*.

**Conclusion:**

Our study has characterized the largest contiguous stretch of physically mapped sex chromosome sequence (352 kb) from a ZZ/ZW lizard species. Although this region represents only a small fraction of the sex chromosomes of *P. vitticeps*, it has revealed several features typically associated with sex chromosomes including the accumulation of large blocks of repetitive sequences.

## Background

Chromosomes are remarkably conserved across vertebrates, showing striking conservation of gene content and gene order of substantial regions of synteny over millions of years. For example, the karyotypes of different bird species are extremely similar [1] and comparative chromosome painting of squamate reptiles (lizards and snakes) macrochromosomes shows ancestral homology dating back to the early Jurassic [2,3].

In remarkable contrast to this deep conservation, and surprising, considering their critical role in reproduction, sex chromosomes show a lack of homology and frequent rearrangements and transitions between and even within lineages [4]. The most stable and conserved vertebrate systems are in therian mammals (eutherians and marsupial mammals with a highly conserved XX female: XY male system of male heterogamety), and birds and snakes (with highly conserved ZW female: ZZ male systems of female heterogamety). These three chromosome systems, that is, XY of mammals, ZW of snakes and ZW of birds show no genetic homology and are thought to have evolved independently [4,5]. The evolutionary dynamism of sex chromosomes is more obvious from the distinct forms of XY and ZW chromosomes among vertebrates even within taxonomic families [5,6]. It is this lability and the role of the sex chromosomes in the fundamental trait of sex determination that makes non-mammalian vertebrates of extreme interest for studying the processes behind genome organization and evolution [5-8].

Sex chromosomes are generally thought to have evolved from a homologous autosomal pair, when one partner acquired a sex determining gene [9-11]. Accumulation of sexually advantageous mutations near the sex determining locus led to selection for suppression of recombination, which in turn accelerated the loss of active genes, deletions and insertions from the sex specific Y or W chromosomes [11-14]. The loss of genetic material and accumulation of repetitive sequences on the Y or W is obvious in many species as cytological heteromorphism. This process of repeat accumulation is rapid and stochastic, generating various intermediates between homomorphy and extreme differentiation in squamate reptiles (snakes and lizards), and variation in the gene content as can be seen in the Y chromosome of different mammals [6,15].

Despite the discovery of XY and ZW sex chromosomes in the early 19^th^ century [16-18], few master sex determining genes have been identified. These include *SRY* in therian mammals [19,20], and a variety of genes in fish and frog: *dmy* in medaka (*Oryzias latipes*) [21,22], *gsdf(Y)* in medaka (*O. luzonensis*) [23], *dmw* in clawed frog (*Xenopus laevis*) [24], *sdy* in rainbow trout (*Oncorhynchus mykiss*) [25], and *amhy* in Patagonian pejerrey (*Odontesthes hatcheri*) [26]. In all but three cases (*sdy* in rainbow trout, *amhy* in Patagonian pejerrey and *gsdf(Y)* in medaka) [23,25,26], master sex determining genes are transcription factors, in so far as they appear to act by up- or down-regulating transcription in the vertebrate sex differentiation pathway. This newly discovered diversity in regulation suggests that the *de novo* evolution of master sex determining genes from genes that are not involved in sex differentiation can occur in vertebrates. It occurs less frequently than those that are involved in the sexual differentiation pathway. However, due to recent advancement in molecular genetics and genome sequencing technologies, novel master sex determining genes are likely to be discovered at an accelerating rate, in turn discovering novel mechanisms of sex determination.

Reptiles are of particular interest because they display astonishing variation in the sex determining system. They are represented by species which display all types of chromosomal system (e.g. XY, ZW, including multiple sex chromosomes) and temperature-dependent sex determination (TSD), as well as species in which the two modes of sex determination interact [6,8,27-29]. This diversity is well established in the literature, so it is remarkable that little is known of the genes involved. Non-avian reptiles remain the only vertebrate group in which no master sex determining gene has been identified. They therefore promise to yield novel insights into vertebrate sex determination.

The best-studied sex chromosomes among non-avian reptiles are those of snakes [30-34]. Like birds, snakes have a ZZ male ZW female system, and Z gene content is conserved across all species so far examined. However, gene mapping shows that the sex chromosomes of snakes are not homologous to those of birds or mammals [31,32], nor to those of a ZW turtle (*Pelodiscus sinensis*), a ZW gecko (*Gekko hokouensis*), a ZW dragon lizard (*Pogona vitticeps*) or an XY green anole (*Anolis carolinensis*) [31,32,35-38]. Four genes that are Z-linked in *G. hokouensis* are autosomal in the dragon lizard *Pogona vitticeps*[35] and in snakes [32,37] and five genes that are Z-borne in snakes and birds are autosomal in the dragon lizard *P. vitticeps*[35]. Even within dragons (Agamidae), comparative mapping of a sex-linked marker shows that the ZW sex microchromosomes of three Australian agamid species (*P. vitticeps, P. barbata* and *Diporiphora nobbi*) are homologous, but their homology does not extend to a fourth species, *Ctenophorus fordi*[29,39]. Thus there is likely to be a number of independent origins of the sex chromosomes, not only between two families of lizards (Gekkonidae and Agamidae), but also among closely related species in the same subfamily.

The central bearded dragon, *P. vitticeps*, is a powerful model for the study of the evolution of sex determination because it has differentiated sex chromosomes [40], but phenotypic sex can be reversed by high temperatures, producing all female individuals [28]. Thus, sex in *P. vitticeps* is determined by the interplay between genotype and environment, producing genotype-phenotype discordant individuals. Discovering novel sex determining genes in this species will therefore broaden our understanding of how sex is genetically determined, as well as of how environment can regulate genetic machinery. This species has 16 pairs of chromosomes – 6 pairs are macrochromosomes and 10 pairs are microchromosomes [41] – with female heterogamety (ZZ male, ZW female) [40]. The sex chromosomes are microchromosomes and the W chromosome is larger than the Z [42]. The W chromosome is also highly heterochromatic [40] which suggests that its sequence content is different from the Z and that an amplification of repetitive DNA is likely to have occurred during its evolution.

We previously reported the isolation of Z and W linked markers, developed a genetic sex test and sequenced a 3.2 kb region of the sex chromosomes [28,29] in *Pogona vitticeps*. In this study, we extend the sequenced region of the *P. vitticeps* sex microchromosomes to 352 kb using a BAC walking approach. We report 28 open reading frame (ORFs) including three gene family members (*oprd, rcc and znf*), and eight major groups of repetitive elements. Comparative analysis of this partial sex chromosome sequence, allowed us to identify additional potential candidate sex chromosome genes in *P. vitticeps*. One of these genes, *R-spondin 1* (*rspo1*), has a central role in vertebrate ovarian differentiation [43-45] and we evaluate it as a candidate sex-determining gene in *P. vitticeps*.

## Results

### Isolation and physical mapping of *Pogona vitticeps* sex chromosome BAC clones

**Figure 1:**
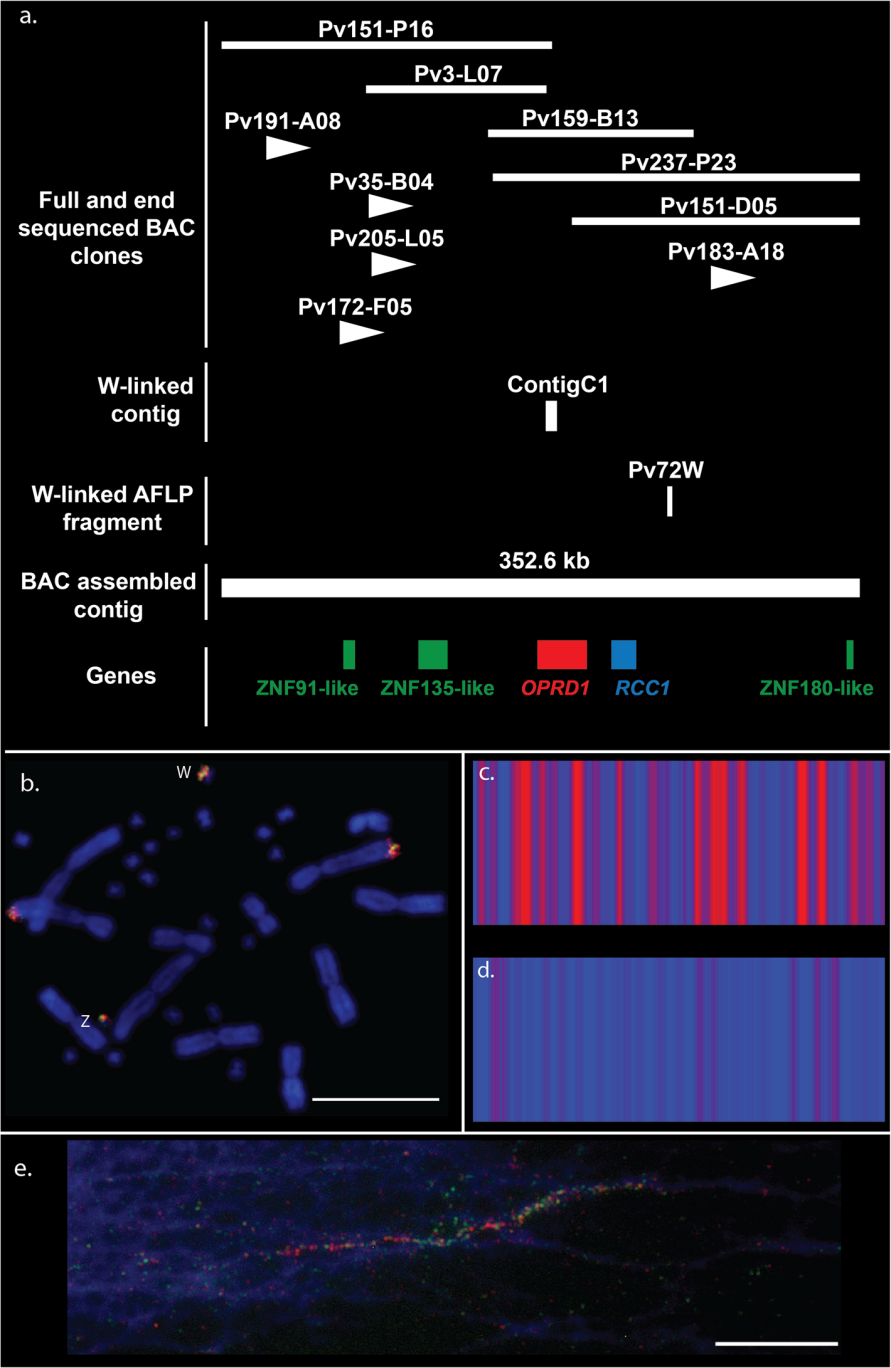
**Isolation and physical mapping of sex chromosome BAC clones in *Pogona vitticeps*. a**: schematic representation of 352 kb partial sex chromosome contig showing relative locations (not to scale) of fully and end sequenced BAC clones, previously identified W-linked contig and AFLP marker, proteins and genes; **b**: two colour FISH showing locations of two sex chromosome BAC clones at either end of the *P. vitticeps* 352 kb contig (Pv151_P16 green and Pv151_D05 orange; yellow denotes overlapping signals). Hybridization signal differences between Z and W chromosomes are visible; **c**: scalable vector graphics (SVG) plot (generated by Repbase-GIRI [48]) of sex chromosome BAC clone Pv03_L07, high density and frequency of red vertical bars represent distributions and locations of repetitive sequences; **d**: SVG plot of autosomal BAC clone Pv176_G09 showing low frequencies of red vertical bars indicating accumulation of low number of repeats compared to that of the sex chromosome BAC clone; **e**: two colour fiber FISH (Pv151_P16 orange, Pv237_P23 green), showing orange and green regions and regions of overlap (yellow). Scale bars represent 10 μm.

Ten BAC clones were isolated by genomic library screening with previously identified sex linked sequences [27,28] and chromosome walking. We sequenced five of the 10 BAC clones, which ranged in length from 98 kb to 202 kb (GenBank Accessions: KF541650; KF541651; KF541652; KF541653; KF541655, Table 1), while the remaining five BAC clones overlapped the sequenced clones. Reads from all five BAC clones were assembled into a single contiguous sequence of 352 kb (GenBank Accession: KF541658). Analysis of this partial sex chromosome sequence revealed that the contig contains the previously identified W marker Pv72W and contig C1, but not the Z marker Pv71Z [27,28] (Figure 1a). This implies that the contig is likely to have W chromosomal origin and the contig therefore represents partial W sequence (Figure 1a). The BAC clones were physically mapped by DNA FISH as well as by fiber FISH to verify their location on the sex chromosomes (Figure 1b, e). The intensity and pattern of hybridisation signals of two colour DNA FISH with BAC clones Pv151_P16 and Pv151_D05 were different between Z and W chromosomes. Primary hybridisation of the BAC clones was to the Z and W chromosomes, with signal on the W chromosome more intense than that on the Z chromosome (Figure 1b). Terminal hybridisation signals on the long arm of chromosome 2 suggested these BAC clones may share some repetitive elements with the NOR-associated heterochromatin (Figure 1b, [42]).

**Table 1:** **A comparison of the major classes of repetitive sequences in sex chromosomal and autosomal BAC clones in *Pogona vitticeps***

**Repeat class**	**Sex chromosome BAC clones**										**Autosome BAC clone**					
	**Pv03_L07 (98 kb)**		**Pv151_P16 (183 Kb)**		**Pv159_B13 (113 Kb)**		**Pv237_P23 (202 kb)**		**Pv151_D05 (156 Kb)**		**Pv176_G09 (145 Kb)**		**Pv100_O13 (139 kb)**		**Pv215_D15 (134 Kb)**	
	**Fragments**	**Size (bp)**	**Fragments**	**Size (bp)**	**Fragments**	**Size (bp)**	**Fragments**	**Size (bp)**	**Fragments**	**Size (bp)**	**Fragments**	**Size (bp)**	**Fragments**	**Size (bp)**	**Fragments**	**Size (bp)**
Integrated virus	0	0	0	0	1	47	1	47	0	0	0	0	0	0	0	0
Interspersed repeat	1	49	3	254	2	126	1	49	1	77	2	165	0	0	0	0
DNA transposon	30	2872	53	4050	37	3250	69	5288	58	4343	58	5283	49	6140	40	4906
Endogenous retrovirus	23	5252	39	6836	9	489	16	1036	11	726	25	1888	16	1132	14	990
LTR transposon	37	12385	87	25553	48	9078	82	12342	61	5673	44	3613	66	4350	71	5287
Non-LTR transposon	58	17770	101	28645	74	17366	148	46284	121	40003	46	5767	51	5000	54	5448
Pseudogene	0	0	0		0	0	1	68	1	68	2	173	0	0	0	0
Simple repeat	21	2189	35	3743	6	597	37	3710	29	2941	0	0	2	137	2	133
Total	170	40517	318	69081	177	30953	355	68824	282	53831	177	16889	184	16759	181	16764
Proportion (%)		41.3439		37.74918		27.392		34.0713		34.5071		11.6476		13.9658		10.4775

Two of the largest BAC clones, Pv151_P16 (about 182 kb) and Pv237_P23, (about 202 kb), which represent the two ends of the 352 kb contig and overlap in the middle, showed green, orange and yellow (overlapping) fluorescent signals on chromatin fibers (Figure 1e) confirming our physical map of BAC clones (Figure 1b). The observation of multiple regions of green and orange on a single fiber suggests repetition of sequences contained within the BAC outside the contig (Figure 1e). As a control for comparative analysis between sex chromosome and autosome sequences, substantial genomic sequences were also obtained from three autosomal BAC clones (Table 1).

### Sequence analysis of the partial sex chromosome contig

We used Genscan [46] to analyze sequence of this contig. This identified the contig as an isochore type 2 with 44.5% GC content. Genscan also predicted 28 ORFs (Table 2). They ranged between 57 amino acids (aa) to 3194 aa (Table 2). BLAST (blastp, *Anolis carolonensis*; [47]) analysis of these ORFs supported the predictions for the protein-coding genes, opioid receptor, delta 1 (*oprd1*) and regulator of chromosome condensation 1 (*rcc1*) and three zinc finger protein genes, *znf91*, *znf135* and *znf180* (Table 2).

**Table 2:** **Summary of Blastp (refseq *Anolis carolinensis*** [47]**) analysis of Genscan**[46]**predicted ORFs from 352 kb contig sequences in *Pogona vitticeps***

**ORF**		**Predicted genes/proteins**	**Total score**	**Query cover**	**E value**	**Max identity**	**Accession anolis**
**No.**	**Length (aa)**						
1	776	PREDICTED: receptor expression-enhancing protein 3-like	94.4	12%	SE-21	55%	XP_003223495.1
2	2208	PREDICTED: hypothetical protein in LOC100567032	302	27%	3E-82	32%	XP_00322950.1
3	576	PREDICTED: protein FAM135-B like	33.5	18%	0.33	26%	XP_003219535.1
4	718	PREDICTED: zinc finger protein 91-like	9847	83%	0	78%	XP_003229097.1
5	978	PREDICTED: retrotransposable element Tf2 155 kDa protein type 1-like	423	82%	1E-83	36%	XP_003224123.1
6	92	PREDICTED: leucine-rich repeat and fibronectin type III domain-containing protein	27.7	54 %	0.65	34 %	XP_003222916.1
7	1575	PREDICTED: retrotransposable element Tf Tf2 155 kDa protein type-like	360	40%	3E-79	39%	XP_003220874.1
8	340	PREDICTED: 1, 25-dihydroxivitamin D(3) 24-hydroxylase, mitochondrial-like	113	37 %	2E-27	48 %	XP_003228807.1
9	1092	PREDICTED: zinc finger protein 135-like	3327	41%	0	73%	XP_003229099.1
10	1012	PREDICTED: hypothetical protein LOC100556889	155	26%	7E-39	34%	XP_003217306.1
11	1009	PREDICTED: retrotransposable element Tf2 155 kDa protein type 1-like	364	65%	7E-81	40%	XP_003220874.1
12	204	PREDICTED: serologically defined colon cancer antigen 8-like	57	45%	9E-10	40%	XP_003225236.1
13	298	PREDICTED: 1, 25 -dihydroxyvitamin D(3) 24-hydroxylase, mitochondrial-like	68.6	42%	5E-13	34%	XP_003228807.1
14	573	PREDICTED: hypothetical protein LOC100566709	253	43%	5E-73	48%	XP_003227941.1
15	297	PREDICTED: RNA-binding protein with multiple splicing-like, partial	31.2	8%	0.41	56%	XP_003227406.1
16	418	PREDICTED: delta-type opioid receptor-like	623	90%	0	82%	XP_003229441.1
17	118	PREDICTED: leucine-rich repeat neuronal protein 2-like	28.5	41%	0.94	26%	XP_003220412.1
18	125	PREDICTED: ubiquitin-conjugating enzyme E2 E1-like isoform 1	29.3	52%	0.42	28%	XP_003226269.1
19	106	PREDICTED: LOW QUALITY PROTEIN: dynein heavy chain 17, axonemal-like	28.9	81%	0.53	28%	XP_003217173.1
20	424	PREDICTED: regulator of chromosome condensation-like	734	100%	0	85%	XP_003229442.1
21	748	PREDICTED: ras-related protein Rab-17-like	113	18%	3E-27	44%	XP_003215266.1
22	57	PREDICTED: class I histocompatibility antigen, F10 alpha chain-like	28.1	50%	0.21	31%	XP_003227795.1
23	527	PREDICTED: hypothetical protein LOC100561123	93.2	49%	1E-19	28%	XP_003229050.1
24	253	PREDICTED: excitatory amino acid transporter 2-like	28.1	22%	4.5	41%	XP_003226244.1
25	3194	PREDICTED: hypothetical protein LOC100561123	546	33%	3E-39	29%	XP_003225516.1
26	358	PREDICTED: ubiquitin carboxyl-terminal hydrolase 25-like	67.4	74%	3E-12	25%	XP_003219106.1
27	573	PREDICTED: hypothetical protein LOC100567032	53.9	14%	2E-07	33%	XP_003229050.1
28	396	PREDICTED: zinc finger protein 180-like, partial	1130	44%	3E-79	72%	XP_003230299.1

Only the top hits based on E-values are listed.

The repetitive sequence content of sex chromosomes was examined using Repbase-GIRI [48]. Analysis of repetitive sequences of the sex chromosome BAC clones revealed the highly repetitive nature of *P. vitticeps* sex chromosome sequences (Figure 1c and d, Table 1, Additional file 1). For example, one of the smallest sex chromosome BAC clones, Pv03_L07 (about 98 kb) contains 41% repetitive sequences of which 43% are non-LTR retrotransposons (Table 1). Overall, LTR and non-LTR retrotransposons constituted the bulk of the repetitive sequences of the sex chromosome BAC clones. We compared this composition with that of three autosomal BAC contigs. In general, sex chromosome BAC clones have three times more repetitive sequences (Table 1). Autosomal BAC clones showed no preferential accumulation of any specific repetitive sequences (Table 1).

We also investigated the accumulation of microsatellite repeats within the 352 kb sex linked contig using Msat commander [49]. Our analysis identified 16 STR loci consisting of three di-nucleotide (AC, AG, AT), three tri-nucleotide (AAC, AGC, AAT), eight tetra-nucleotide (AAGG, AATC, AATG, ACGC, AAAC, ATCC, AGAT, AAAT), and two penta-nucleotide (AAAAT, AAATC) repeat motifs within the 352 kb sex linked contig. At least five of these loci were found to be amplified on the W chromosomes (data not shown, will be published elsewhere). These loci therefore provide opportunities to develop sex-linked markers.

### Comparative analysis of the sequenced region

Comparative orthology analysis revealed that orthologues of *oprd1* and *rcc1* are part of a conserved homologous syntenic block (HSBs) in other vertebrates (e.g. chicken, chromosome 23; human chromosome 1). This region is not on the sex chromosomes of these other species, confirming the independent origin of the dragon sex chromosomes.

Analysis of genes in this HSB identified a candidate sex determining gene for *P. vitticeps*, the mammalian ovarian differentiation gene *R-spondin 1*(*RSPO1*). Since, we did not find *rspo1* sequences within the sequenced region, we cloned and mapped the dragon *rspo1* orthologue.

### Cloning and characterization of *rspo1*

**Figure 2:**
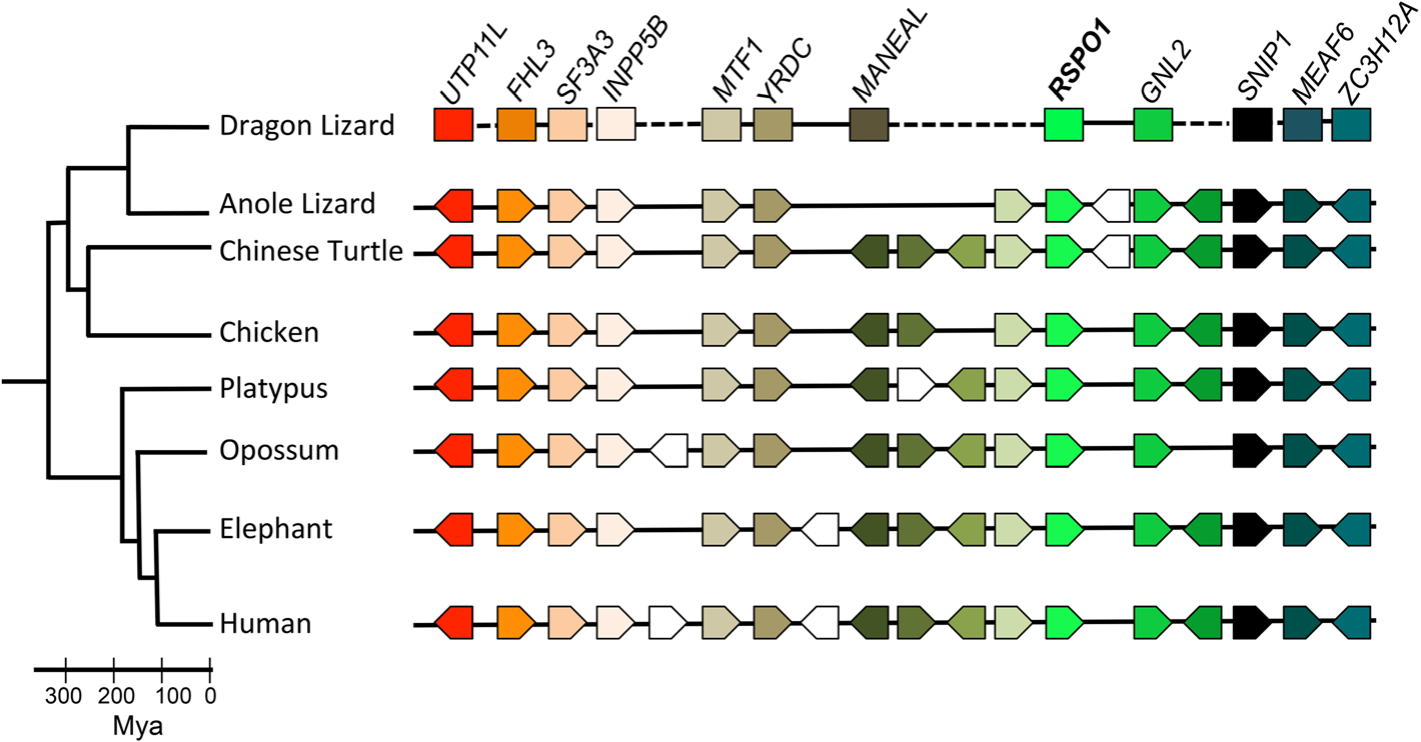
**Gene content of the sequenced *rspo1-*containing BAC clones in the dragon lizard (*Pogona vitticeps*) is conserved when compared to other amniote species.** Arrows indicate direction of transcription. Gene order and orientation is unknown for the dragon lizard due to gaps in BAC sequence. White arrows indicate genes with no orthologue in the region for the other species shown.

We screened the BAC library using primers designed from sequences obtained from a testis transcriptome (T. Ezaz unpublished). Using this approach we isolated two *rspo1-*containing BAC clones (Pv100_O13 and Pv215_D15; GenBank Accessions: KF541656; KF541657). These BAC clones were annotated using Genscan [46], revealing 19 and 17 ORFs respectively (Additional file 2). Blastp [47] analysis of those ORFs identified 10 genes from the region surrounding *rspo1* in other amniote species; Figure 2, Additional file 2), corresponding to the conserved HSB homologous to chicken chromosome 23 and human chromosome 1.

We also cloned cDNA of a homologue of *rspo1* in *P. vitticeps* by PCR amplification of RNA using primers designed against *P. vitticeps’* testis transcriptome derived *rspo1* (T. Ezaz unpublished). We sequenced three cDNA clones, finding consensus sequences of the two fragments 703 and 956 bp length which partially overlapped. The total length of the consensus sequence covered by the two fragments was 1347 bp.

To eliminate the possibility that *rspo1* gene sequences reside on sex chromosomes in this species, we directly sequenced the PCR products from 10 phenotypic males and 10 phenotypic females. Direct PCR product sequencing was interrupted by the presence of a polymorphic imperfect pentanucleotide repeat (ACCCC_n_) in the intronic region between exon 3 and exon 4 (data not shown). The successfully sequenced regions comprised between 9.6% and 94.4% of the amplicon, although those regions uniformly had a high percent identity with the *rspo1* genomic reference (98.1% - 100% pairwise percent identity) from two fully sequenced BAC clones (Pv100_O13 and Pv215_D15). To circumvent this problem we cloned and sequenced PCR products and confirmed the *rspo1* sequence identity for the full length of the amplicon (Additional file 3).

### Mapping *rspo1*

**Figure 3:**
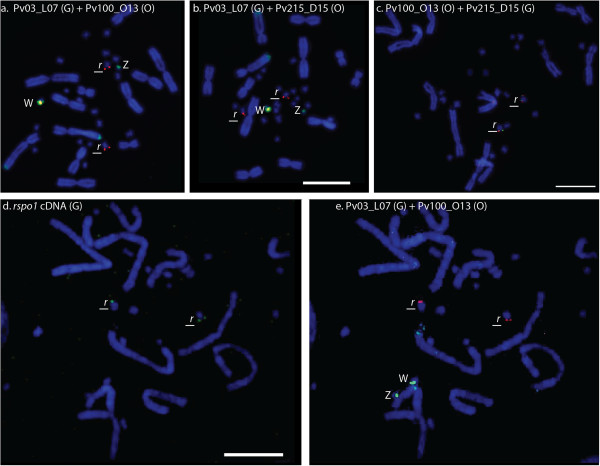
**Physical mapping of BAC and cDNA clones containing *rspo1 *gene in female *Pogona vitticeps*. a**: merged image showing locations of BAC clones Pv03_L07 (green) and Pv100_O13 (orange) containing *rspo1*; highly amplified signals from the sex microchromosome BAC clone Pv03_L07 was observed on the W microchromosome, while clear signals from both *rspo1* BAC clones were observed on a different pair of microchromosomes. Note that a yellow signal (overlap of orange and green fluorochromes) is observed on W chromosome, suggesting hybridization of shared repetitive sequences from both BAC clones containing sex chromosome sequences and *rspo1* gene sequences. BAC clone Pv03_L07 hybridised onto *P. vitticeps* sex chromosomes and telomeric region of long arm of chromosome 2; **b**: merged image showing locations of BAC clones Pv03_L07 (green) and Pv215_D15 (orange) containing *rspo1,* which had the same pattern as the BAC clone Pv100_O13 (Figure 3a); **c**: merged image of two *rspo1* BAC clones Pv215_D15 (green) and Pv100_O13 (orange), showing co-locations of both BAC clones on the same pair autosomal microchromosomes. Note the absence of hybridisation signal on the W chromosome (3a,b) which was suppressed by species specific c0t-1 DNA **d**: hybridization signal of *rspo1*cDNA clone in female metaphase chromosome spreads; **e**: two colour FISH on the same slide as panel d, showing mapping of *P. vitticeps’* sex chromosome BAC clone Pv03_L07 (green) with BAC clone Pv100_O13 (orange) containing *rspo1*. Hybridisation signals of sex chromosome BAC clone and the *rspo1* cDNA clone are not on the W chromosomes, suggesting that *rspo1* is autosomal in *P. vitticeps*. The hybridization signal on the W chromosome from BAC clone FISH is a result of hybridization of shared repeat sequences contained in those BAC clones. G = green; O = Orange; r = *rspo1*; W = W chromosome; Z = Z chromosome. Scale bars represent 10 μm.

To determine if *rspo1* lies with *oprd1* and *rcc1* on the sex chromosomes in *P. vitticeps*, we mapped two BAC clones (Pv100_O13 and Pv215_D15) containing *rspo1*. Each BAC clone was physically mapped on to *P. vitticeps* metaphase chromosome spreads along with a *P. vitticeps* sex chromosome specific BAC clone, Pv03_L07 (Figure 3a,b). These two colour FISH experiments (Pv03_L07 + Pv100_O13 and Pv03_L07 + Pv215_D15) showed hybridisation signals on the centromeric region of the W chromosome in females (yellow signal in Figure 3a,b), as well as a single pair of microchromosomes in both males and females (Figure 3a,b, only female patterns are presented in Figure 3).

Since this pattern could have been produced by shared repetitive sequences, we carried out a second round of FISH with the *rspo1* positive BAC clone Pv100_O13, the sex chromosome BAC clone Pv03_L07, and *rspo1* cDNA clone (GenBank Accession: KF541659) on the same chromosome preparations. The cDNA clone mapped only a pair of microchromosomes (Figure 3d). Because of the small size of the cDNA clone (about 1.3 kb), the hybridisation signal strength on chromosomes was weak and the frequency of hybridisation signals per cell was also low. Only about 30% metaphase cells had signals and signals were usually on only one chromosome, which is usually common in FISH mapping of cDNA clones [50]. The autosomal microchromosome to which *rspo1* cDNA clones mapped was identified by co-hybridisation with BAC clone Pv100_O13. No metaphase cell had *rspo1* cDNA signals on sex microchromosomes identified by clone Pv03_L07 (Figure 3e). Thus evidence from the hybridisation of cDNA clone points to *rspo1* residing on an autosomal microchromosome pair and not on the sex chromosomes. In addition, we performed two colour FISH using two *rspo1* BAC clones (Pv215_D15 + Pv100_O13) with male *P. vitticeps’* specific c0t-1 DNA as suppressor DNA. Both BAC clones were hybridised onto the same pair of microchromosomes identifying their colocations (Figure 3c). We did not observe any centromeric hybridisation signals on the W chromosomes, suggesting repetitive sequences were successfully suppressed by c0t-1 DNA, which also confirm the cDNA clone mapping, i.e. *rspo1* is autosomal.

### Rearrangement of the *rcc1-oprd1-rspo1* region in *Pogona vitticeps*

**Figure 4:**
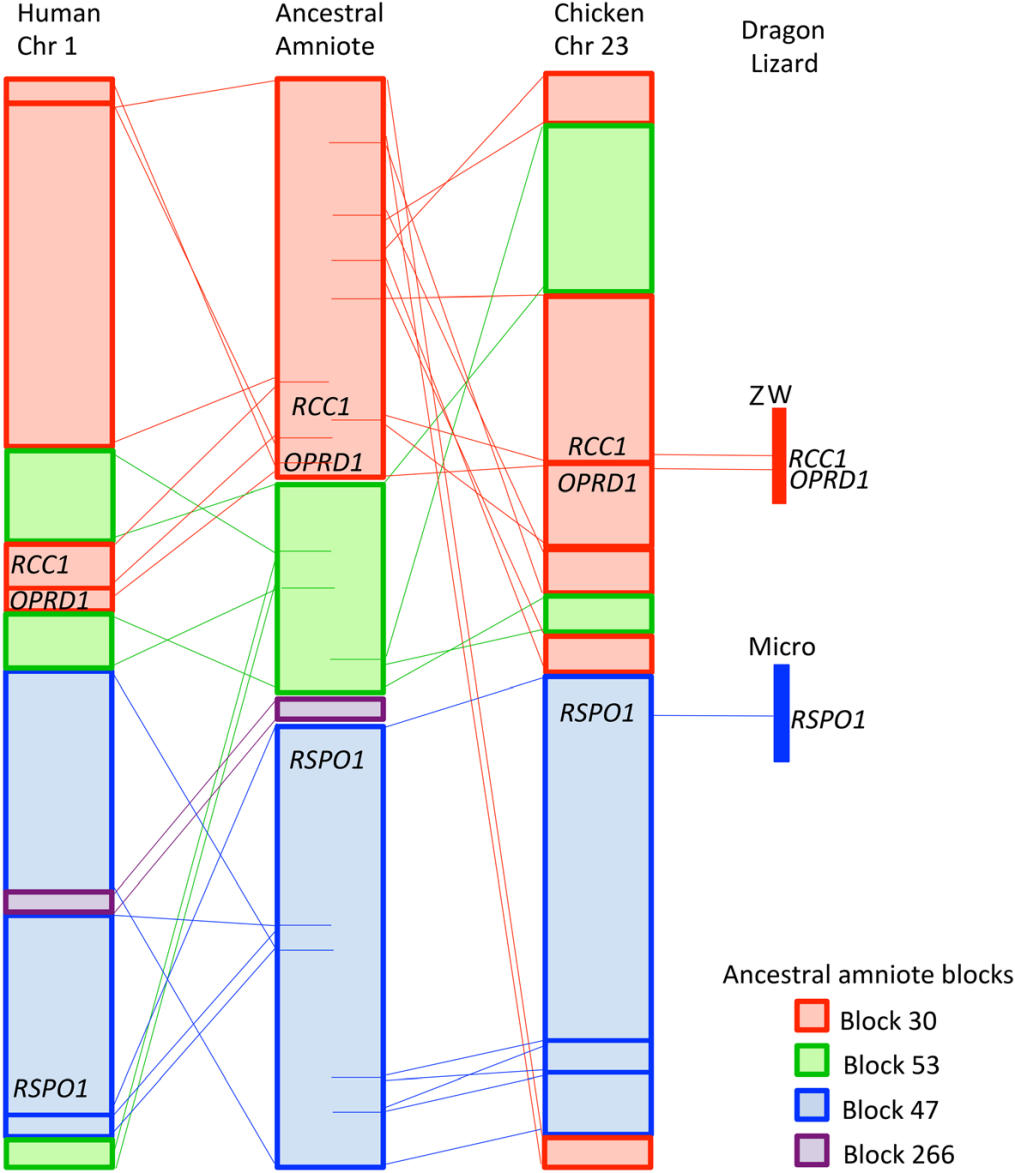
**Comparison of conserved gene block arrangement for the region encompassing genes *rcc1, oprd1 *and *rspo1 *on human chromosome 1 and chicken chromosome 23 with the ancestral amniote arrangement predicted in the Genomicus database (ancestral amniote block numbers refer to block numbers in the Genomicus database) and *Pogona vitticeps*.** Horizontal lines shown within the ancestral amniote blocks indicate breakpoints. Lines connecting the amniote ancestor either human chromosome 1 or chicken chromosome 23 indicate relative position of genes between species.

A closer examination of the region encompassing *rcc1*, *oprd1* and *rspo1* indicated that this region was rearranged during amniote evolution. Although the gene content of this region is conserved between human chromosome 1 and chicken chromosome 23, the Genomicus database [51] has placed *rcc1* and *oprd1* on a putative amniote ancestral block different from that harboring *rspo1.* A number of different breakpoints are observed in the *rcc1/oprd1* region when gene arrangements of the predicted ancestral blocks are compared to human chromosome 1 and chicken chromosome 23 (Figure 4).

The two BAC clones containing *rspo1* (Pv100_O13 and Pv215_D15) also contained 10 genes from the region surrounding *RSPO1* in other amniote species, but the sequences from these two BACs do not assemble well enough to determine whether gene arrangement is also conserved between *P. vitticeps* and other amniotes.

## Discussion

Our study has characterized the largest contiguous stretch of physically mapped sex chromosome sequence (352 kb) from a ZZ/ZW lizard species. Although this region represents only a small fraction of the sex chromosomes of *P. vitticeps*, it has revealed several features typically associated with sex chromosomes including the accumulation of large blocks of repetitive sequences.

The 352 kb of sex chromosome sequence that we have identified in this study showed large fold accumulation of LTR and non-LTR retrotransposons including CR1, and LINEs (including BovB, L1, L2). Accumulation of such repetitive sequences are common features on sex chromosomes and have been reported for the sex chromosomes of many vertebrate species from insects to mammals [52-60]. In particular, these repeats have been found to be preferentially accumulated on the sex chromosomes with BovB and CR1 repeats found on the mouse Y chromosomes and the chicken Z chromosomes respectively. It appears likely therefore, that these repeats may have converged, preferentially on the sex chromosomes in a number of taxa [33] perhaps acting as an evolutionary driver for the degeneration of sex chromosomes in vertebrates.

Our sequencing and physical mapping strategy revealed two known protein coding genes in addition to three zinc finger genes, within the sequence on the *P. vitticeps* sex chromosomes. The gene *oprd1* is a member of G-protein-coupled receptors, which constitute a vast protein family that performs a wide range of functions (including various autocrine, paracrine and endocrine processes) [61,62]. While the gene *rcc1* is a eukaryotic protein which binds to chromatin and interacts with RAN enzyme, a nuclear GTP-binding protein, to promote the loss of bound GTP and the uptake of fresh GTP, thus acting as a guanine-nucleotide dissociation stimulator (GDS) [63]. The interaction of *rcc1* with RAN probably plays an important role in the regulation of gene expression. Neither of these two genes is known to be involved with sex determination or gonad differentiation of other species and we have no direct evidence that *oprd1* and *rcc1* are involved in the *P. vitticeps* sex determination pathway. Both are expressed in a range of adult mammalian tissues (NCBI UniGene, [64]). Thus these genes, though on the sex chromosomes of *Pogona vitticeps*, are unlikely candidates for a sex determination function.

Orthologues of *oprd1* and *rcc1* are located on a syntenic block that is conserved among amniotes (Figure 4). This synteny suggested an evolutionarily conserved arrangement, which would allow us to identify other genes on the *P. vitticeps* sex chromosomes. One of the genes in this conserved syntenic block, *rspo1*, is involved in ovary differentiation in mammals. This gene was discovered by its mutation in a family with four XX males, and appears to work by activating beta-catenin pathway [43,65]. It was therefore a good candidate for a role in gonad differentiation in *P. vitticeps.* However, it is unlikely to be a candidate for the master sex determining switch as our cDNA clone and two colour BAC FISH mapping has unequivocally established its location on a single pair of autosomal microchromosomes. Although, BAC clones containing *rspo1* sequence mapped to the W chromosome as well as an autosomal microchromosome, this is presumably because they contain repeats shared with the sex chromosomes, which can be suppressed by species specific suppressor DNA (Figure 3c). The absence of *rspo1* from the sex chromosomes eliminates this candidate gene from consideration. Importantly, comparative sequence analysis of this newly characterized *P. vitticeps* partial sex chromosome sequence showed no homology to genes or sequence on other vertebrate sex chromosome, implying that the ZW sex chromosomes in this species evolved independently [35].

Evolution of the dragon sex chromosomes seems to have involved genome rearrangements (Figure 4). The 352 kb contig contains only two genes (*oprd1* and *rcc1*) corresponding to chicken chromosome 23, and they are surrounded by zinc finger genes, which are not present in other amniote species. Intriguingly, *P. vitticeps* is not the only species for which a gene from the ancestral amniote specific synteny block containing *oprd1* and *rcc1* has ended up on a sex chromosome. For example, *med18*, a gene adjacent to *rcc1* in the predicted ancestral block, is located on the chicken Z chromosome. Is this merely coincidence, or is there a sequence in this region that has a selectable function in sex determination? In contrast, BAC clones containing *rspo1* contain a number of genes found adjacent to this gene in other species. We predict that most of the chicken chromosome 23 synteny block will reside on the same microchromosome pair in *P. vitticeps* as *rspo1* and not on the sex chromosomes.

## Conclusions

Our study has demonstrated that the sex chromosomes of *P. vitticeps* share some characteristics of sex chromosomes observed in other vertebrate taxa, namely an accumulation of repetitive elements. However the *P. vitticeps* sex chromosomes also revealed new aspects of sex chromosome evolution that may inform us generally about the rapid evolution of novel sex chromosomes. Of particular interest is our observation that a highly conserved syntenic region that contains *rspo1* was disrupted prior to the formation the *P. vitticeps* sex chromosomes. This work provides the fundamental basis and anchor point from which we can walk to characterize the rest of the dragon W chromosome, and discover a putative sex determining gene(s).

## Methods

### Isolation and sequencing of BAC clones

A genomic BAC library (6.2X) for *P. vitticeps* was screened using an overgo-based protocol [35,66]. Overgos were designed against previously identified sex-linked sequences (GenBank Accessions: EU938138.1; EU938139.1; EU938136.1), which were extended from a 50 and a 49 bp W and Z-linked AFLP fragment (Figure 1 for BAC ID) [28,29]. Initial library screening identified five putative sex linked BAC clones. Five more putative sex-linked BAC clones were identified by subsequent BAC walking. We have also used 7-plate PCR matrix of BAC library to isolate BAC clones containing *rspo1*, following manufacturer’s instruction (Amplicon Express, USA). The library was screened using primer pairs (forward: 5′ CAACTGTGAGGACTGTTTCAGC 3′; reverse: 5′ AAATCACCTGCAGGATCCAC 3′) designed against a testis transcriptome (T. Ezaz unpublished). Primers were designed to amplify a short genomic fragment between *rspo1* exons 4 and 5 of this gene in *P. vitticeps*. The PCR conditions was as follows: an initial denaturation at 95°C for 5 min, followed by 35 cycles of 95°C for 30 s, 62°C for 30 s and 72°C for 90 s; and finally 72°C for 10 min for a final extension.

The full BAC sequences and BAC end sequences were obtained through a commercial vendor (Macrogen, Korea) and Biomolecular Resource Facility (Australian National University, Canberra, Australia) using Sanger and 454 sequencing platforms. In total five sex chromosome BAC clones and three autosomal BAC clones were sequenced (Table 1) using either Sanger or 454 platform. BAC clones were also subjected to end sequencing using pCC1 epicenter vector specific forward and reverse primers: pCC1™ / pEpiFOS™ forward sequencing primer (5′ GGATGTGCTGCAAGGCGATTAAGTTGG 3′) and pCC1™ / pEpiFOS™ reverse sequencing Primer (5′ CTCGTATGTTGTGTGGAATTGTGAGC 3′; Invitrogen). We assembled each BAC using the default parameters of Newbler (V2.6, Roche CT, USA), with an expected coverage of 40X and a custom filtering database that contained the BAC vector and host genome (DH10B). Sequences shared by the individually assembled BAC clones indicated that they were contiguous, so we repeated the assembly with reads from all five BAC clones and mapped reads back to the resulting 352 kb contig. Reads from each BAC occupied a discrete segment of the contig in proportion to the size of the insert, as determined by individual BAC assemblies. Sequences were annotated using Genscan [46] and predicted ORFs then subjected to homology search using Blastp (refseq *A. carolinensis*[47]). The 352 kb contig was also analyzed for homology using Blastn (nr).

### Fluorescence in situ hybridisation (FISH)

#### BAC clone FISH

The chromosomal locations of isolated sex chromosomal BAC clones were verified by physical mapping using FISH following protocols described in Ezaz *et al.*[35]. Briefly, 0.5-1 μg of BAC DNA was labeled by nick translation incorporating directly labeled orange or green dUTP (Abbott Molecular, Botany, NSW, Australia). The labeled BAC DNA was ethanol precipitated, resuspended in 20 μl hybridisation buffer (50% formamide, 10% dextran sulfate, 2X SSC, 40 mmol/L sodium phosphate pH7.0 and 1X Denhardt’s solution), denatured at 68.5°C and hybridised onto metaphase chromosomes spreads overnight at 37°C. Slide washing and microscopy was performed following the protocol described previously by Ezaz *et al*. [35].

#### Fiber FISH

Fiber FISH was performed following a protocol described by Heng *et al*. [67] with modifications to further understand the repeat contents of these BAC clones as well as their arrangements on the sex chromosomes. Briefly, chromatin fiber was obtained by treatment with lysis buffer (0.5% SDS, 50 mM EDTA, 200 mM Tris–HCl pH 7.4). The cell suspension was dropped on slides and air-dried. Slides were soaked in lysis buffer at 90 – 120 s and quickly lifted out to stretch the chromatin fibers and air-dried. After air-drying, the slides were fixed and dehydrated through an ethanol series. The slides were denatured by treatment with 70% 0.1 N NaOH, EtOH 30% (v/v) for 90 s at room temperature. Probe hybridisation, detection and slide analysis was performed following the standard FISH protocol described above.

#### cDNA clone FISH

We cloned the cDNA of a homologue of *rspo1* in *P. vitticeps.* Total RNA was extracted from testicular tissue using RNeasy Plus Universal Mini Kit (Qiagen). The cDNA was synthesized by RT-PCR using Oligo (dT)20 Primer and SuperScript III First-Strand Synthesis System (Invitrogen, Australia), and was used as the PCR template to amplify the homologue of *rspo1*. Two cDNA fragments of the gene were amplified by PCR with the following primer pairs designed against a *P. vitticeps’* testes transcriptome: F1, 5′-AGACAAGCAAGCCAGCAAAC-3′; R1, 5′-CGGACAAGAGGGTAAGCAGA-3′; F2, 5′-CATCCTAGGAGCAGGGCTGT-3′; R2, 5′-CTGGCCACGTCCTTACTG G-3′. The PCR conditions was as follows: an initial denaturation at 95°C for 5 min, followed by 35 cycles of 95°C for 30 s, 55°C for 30 s and 72°C for 90 s; and finally 72°C for 10 min for a final extension. The PCR products were cloned using TOPO TA Cloning Kit (Invitrogen). We sequenced three cDNA clones for respective fragments from a commercial vendor (Macrogen, Korea).

FISH for cDNA clones was performed as described previously by Matsuda *et al.*[50] with slight modification. The DNA probes were labeled using nick translation kit (Roche Diagnostics) incorporating biotin-16-dUTP according to standard protocol. Labeled probes were purified by ethanol precipitation, mixed with hybridisation buffer (50% formamide, 2X SSC, 10% dextran sulfate, 1 mg/ml BSA) and denatured by incubation at 75°C for 10 min. Chromosome slides were denatured in 70% formamide (v/v)/2X SSC for 2 min at 70°C. Approximately, 250 ng of labeled probe (20 μl) per slide was hybridised onto metaphase chromosomes for 1 day at 37°C. Post-hybridisation washes were carried out as following: 50% formamide (v/v)/2X SSC for 20 min at 37°C, 2X SSC for 15 min at room temperature, 1X SSC for 15 min at room temperature and 4X SSC for 5 min at room temperature. The hybridised cDNA probes were reacted with goat anti-biotin antibodies (Vector Laboratories) diluted in 1% BSA/4X SSC for 1 h at 37°C. The chromosome slides were washed as follows: 4X SSC for 5 min at room temperature, 0.1% IGEPAL/4X SSC for 5 min at room temperature, and 4X SSC for 5 min at room temperature. Then the hybridised probes were reacted with Alexa488 rabbit anti-goat IgG (Invitrogen). The chromosome slides were washed using the following series; 4X SSC for 10 min at room temperature, 0.1% IGEPAL/ 4X SSC for 10 min at room temperature, and 4X SSC for 10 min at room temperature. The slides were counter-stained with 20 μg/ml DAPI in 2X SSC and mounted with VectaShield (Vector Laboratories).

### PCR amplification, cloning and sequencing of *rspo1* from multiple *Pogona vitticeps* individuals

To determine the level of polymorphisms at the sequence level between male and female *P. vitticeps*, we screened 20 individuals (10 phenotypic males and 10 phenotypic females). We used the same primer pair to screen the BAC library and followed the same PCR condition. We cloned eight PCR fragments from three males and three females using pGEM-T Easy vector Systems, (Promega) and TOP10 competent cells (Invitrogen). We sequenced the PCR products using M13 and M13R primers using a commercial vendor (Macrogen, Korea).

### Availability of supporting data

All the supporting data are included as additional files.

## Abbreviations

TSD: Temperature-dependent sex determination; GSD: Genotypic sex determination; ORF: Open reading frame; Overgo: Overlapping oligonucleotide probe; BAC: Bacterial artificial chromosome; FISH: Fluorescence *in situ* hybridisation; Contig: Contiguous assembled sequence; cDNA: Complementary DNA; AFLP: Amplified fragment length polymorphism; SNP: Single nucleotide polymorphism; LINE: Long interspersed nuclear element; CR1: Chicken repeat 1 (LINE); GTP: Guanosine-5′-triphosphate; GDS: Guanine-nucleotide dissociation stimulator; BLAST: Basic Local Alignment Search Tool; STR: short tandem repeat (microsatellite).

## Competing interests

The authors declare that they have no competing interests.

## Authors’ contributions

The authors have made the following declarations about their contributions: Conceived, designed and directed the experiments: TE, SDS, AG, JMG. Performed the experiments: TE, DOM, MY, BA, KM, XZ, MJE Contributed analysis tools: TE, AG, DOM, JD, CH; Wrote the paper: TE, JMG, AG, JD, SDS. All coauthors contributed in reviewing the paper. All authors read and approved the final manuscript.

## Supplementary Material

Additional file 1 **Gene content of three autosomal BAC clones.** Sequences were annotated using Genscan [46] and homology search was performed using Blastp [47]. Click here for file

Additional file 2 **Scalable vector graphics (SVG) plot of five sex chromosome and three autosome BAC clones generated by Repbase [**[48]**] showing the distribution of repetitive sequences.** This figure highlights high frequency of red vertical bars representing locations of repetitive sequences in sex microchromosomes compared to low frequencies of red vertical bars on autosomes, which are also microchromosomes. Click here for file

Additional file 3 **Sequence alignment of cloned PCR amplified *rspo1 *** **fragments from three males and females and two BAC clones containing *rspo1*, showing the location of primers and occasional SNPs which are not sex-linked.** Click here for file
