# Supplementary figures and images for: Sequence and gene content of a large fragment of a lizard sex chromosome and evaluation of candidate sex differentiating gene R-spondin 1
Source: BMC Genomics. 2013 Dec 17;14:899. doi: 10.1186/1471-2164-14-899 (PMC3880147; doi:10.1186/1471-2164-14-899)

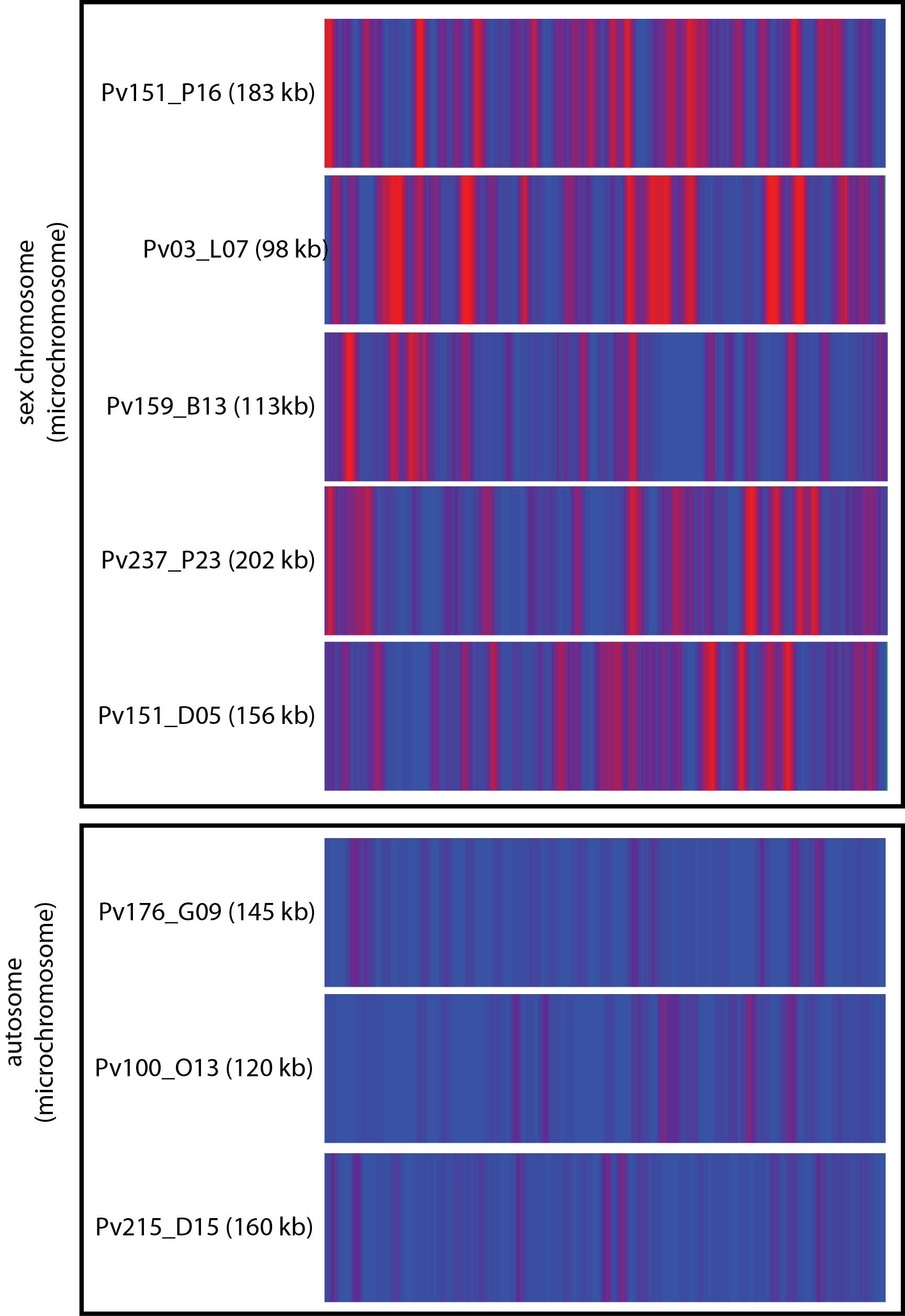

Supplement: Additional file 1 — Gene content of three autosomal BAC clones. Sequences were annotated using Genscan [46] and homology search was performed using Blastp [47]. [file 1471-2164-14-899-S1.png]
