# Supplementary material for: Sequence and gene content of a large fragment of a lizard sex chromosome and evaluation of candidate sex differentiating gene R-spondin 1
Source: BMC Genomics. 2013 Dec 17;14:899. doi: 10.1186/1471-2164-14-899 (PMC3880147; doi:10.1186/1471-2164-14-899)

**Additional file 2**

**Additional file 2:** Gene content of three autosomal BAC clones. Sequences were annotated using Genscan and homology search was performed using Blastp.


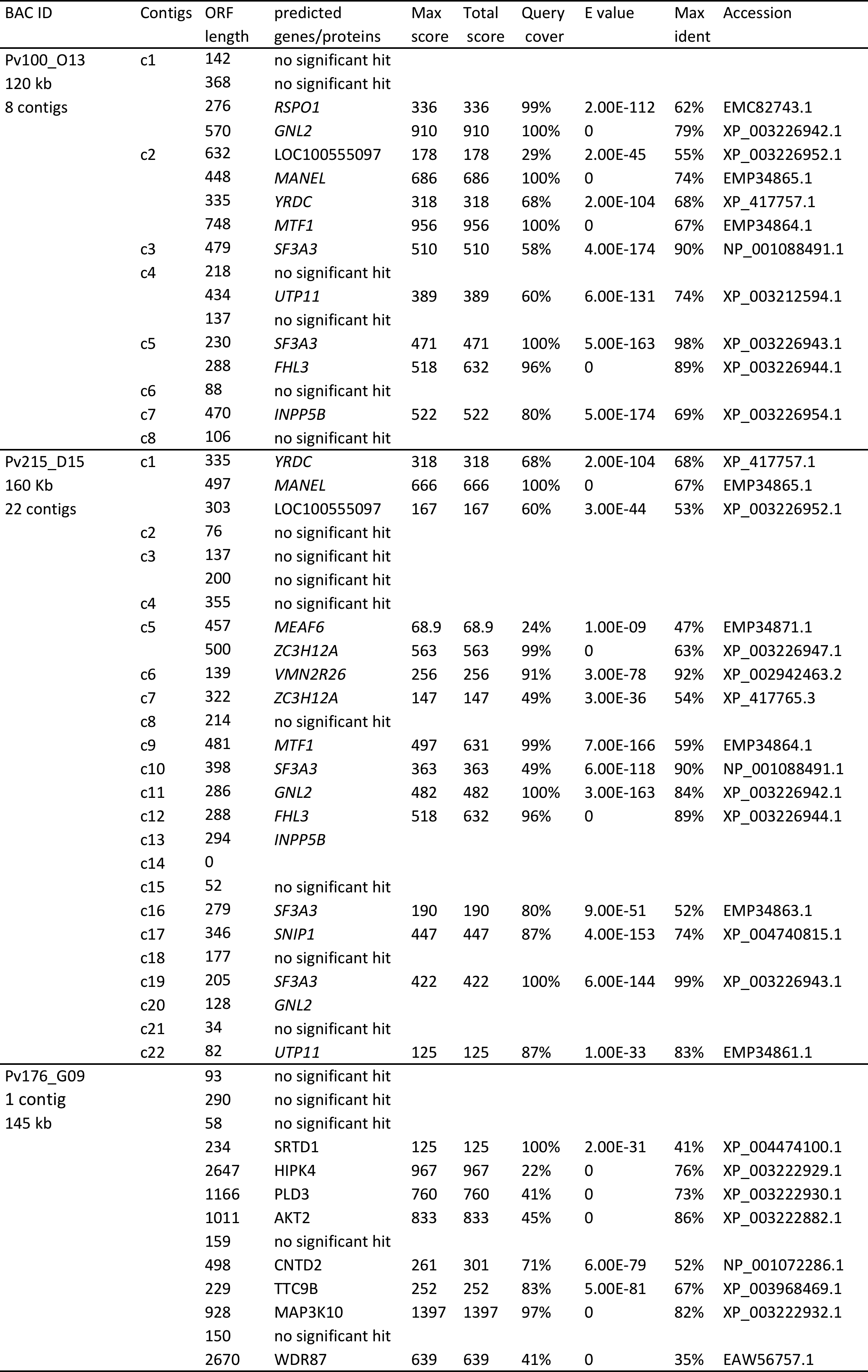

Supplement: Additional file 2 — Scalable vector graphics (SVG) plot of five sex chromosome and three autosome BAC clones generated by Repbase [[48]] showing the distribution of repetitive sequences. This figure highlights high frequency of red vertical bars representing locations of repetitive sequences in sex microchromosomes compared to low frequencies of red vertical bars on autosomes, which are also microchromosomes. [file 1471-2164-14-899-S2.docx]
