# Supplementary material for: Sequence and gene content of a large fragment of a lizard sex chromosome and evaluation of candidate sex differentiating gene R-spondin 1
Source: BMC Genomics. 2013 Dec 17;14:899. doi: 10.1186/1471-2164-14-899 (PMC3880147; doi:10.1186/1471-2164-14-899)
Supplement: Additional file 3 — Sequence alignment of cloned PCR amplified rspo1 fragments from three males and females and two BAC clones containing rspo1 , showing the location of primers and occasional SNPs which are not sex-linked. [file 1471-2164-14-899-S3.pdf]

```
                  1        10        20        30        40        50        60        70        80        90        100       110       120       130
Consensus         CAACTGTGAGGACTGTTTCAGCCGAAACTTTTGCACAAAGTGTAAGGAAGGTTTGTTCTTGCACAAAGGAAGATGTTACGAAACCTGTCCTGAGGGCTTCTCTGCTGCCAACGGCACGATGGAATGCAGCAGTCCGG
                  1        10        20        30        40        50        60        70        80        90        100       110       120       130
1. Pvi RSPO1 Ref  .........................................................................................................................................
                  Primer GF
2. BAC 100-O13                    .........................................................................................................................
3. BAC 215-D15                                       ......................................................................................................
4. Female 1       .........................................................................................................................................
5. Female 2       .........................................................................................................................................
6. Female 3       .........................................................................................................................................
7. Male 1         .........................................................................................................................................
8. Male 2         .........................................................................................................................................
9. Male 3         .........................................................................................................................................

                    140       150       160       170       180       190       200       210       220       230       240       250       260       270
Consensus         GTGAGTGTGGACACGAGGCAGCGAGGGTGGGGTGGG----NGGGTGGCGGTCTGTCTTCTGCTCTCTCTGCCGGCCGGCCTGCCTACTCAGGTCCGCAGTTTGGGTCCCAGGGGCCCATAAGGCAGGTCAGCTCGAC
                    140       150       160       170       176       186       196       206       216       226       236       246       256       266
1. Pvi RSPO1 Ref  ....................................----G...................................................................................T............
2. BAC 100-O13    ....................................----G................................................................................................
3. BAC 215-D15    ....................................----G................................................................................................
4. Female 1       ....................................NNNN.................................................................................................
5. Female 2       ....................................-----................................................................................................
6. Female 3       ................................K...----G................................................................................................
7. Male 1         ....................................----G................................................................................................
8. Male 2         ....................................-----.......R........................................................................................
9. Male 3         ....................................-----...................................A............................................................

                       280       290       300       310       320       330       340       350       360       370       380       390       400       410
Consensus         AGGGTGCTTTCCAAGCCCTTCCCTGCCCAATGGCCCGGAAAGGGCCAYCACGCTCTCCACGTTAGGAGCCTGACTTTCCTGGAACYCCRCATCCCAGTGCCAGGGTCAGTTCGCAAGCTTGGCAGAAACAGGCTCAG
                       276       286       296       306       316       326       336       346       356       366       376       386       396       406
1. Pvi RSPO1 Ref  .........................................A.....C.....................................C..G.........................................A......
2. BAC 100-O13    ...............................................C....G................................T..A................................................
3. BAC 215-D15    ...............................................C....G................................T..A................................................
4. Female 1       .........................................R...........................................C............................................R......
5. Female 2       .........................................R...........................................C............................................R......
6. Female 3       ...............................................C.........................................................................................
7. Male 1         ......................................................................K..............C..A................................................
8. Male 2         ...............................................T.....................................C...................................................
9. Male 3         ...............................................C............R........................C.........................Y..................A......
```

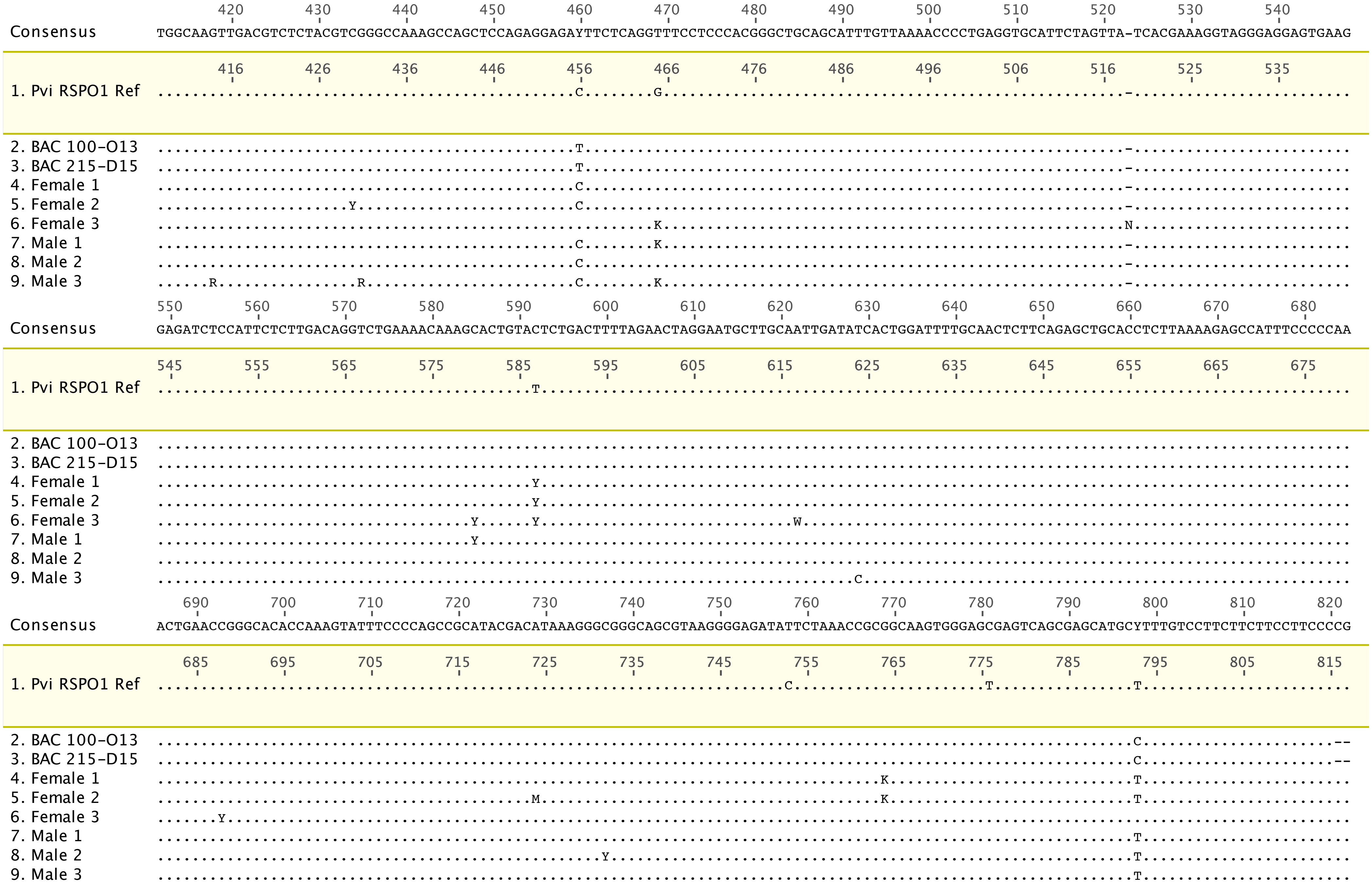
420 430 440 450 460 470 480 490 500 510 520 530 540
Consensus TGGCAAGTTGACGTCTCTACGTCGGGCCAAAGCCAGCTCCAGAGGAGAYTTCTCAGGTTTCCTCCCACGGGCTGCAGCATTTGTTAAAACCCCTGAGGTGCATTCTAGTTA-TCACGAAAGGTAGGGAGGAGTGAAG
416 426 436 446 456 466 476 486 496 506 516 525 535
1. Pvi RSPO1 Ref
2. BAC 100-O13
3. BAC 215-D15
4. Female 1
5. Female 2
6. Female 3
7. Male 1
8. Male 2
9. Male 3
550 560 570 580 590 600 610 620 630 640 650 660 670 680
Consensus GAGATCTCCATTCTCTTGACAGGTCTGAAAACAAAGCACTGTACTCTGACTTTTAGAACTAGGAATGCTTGCAATTGATATCACTGGATTTTGCAACTCTTCAGAGCTGCACCTCTTAAAAGAGCCATTTCCCCCAA
545 555 565 575 585 595 605 615 625 635 645 655 665 675
1. Pvi RSPO1 Ref
2. BAC 100-O13
3. BAC 215-D15
4. Female 1
5. Female 2
6. Female 3
7. Male 1
8. Male 2
9. Male 3
690 700 710 720 730 740 750 760 770 780 790 800 810 820
Consensus ACTGAACCGGGCACACCAAAGTATTTCCCCAGCCGCATACGACATAAAGGGCGGGCAGCGTAAGGGGAGATATTCTAAACCGCGGCAAGTGGGAGCGAGTCAGCGAGCATGCYTTTGTCCTTCTTCTTCCTTCCCCG
685 695 705 715 725 735 745 755 765 775 785 795 805 815
1. Pvi RSPO1 Ref
2. BAC 100-O13
3. BAC 215-D15
4. Female 1
5. Female 2
6. Female 3
7. Male 1
8. Male 2
9. Male 3

```
                 830       840       850       860       870       880       890       900       910       920       930       940     948
Consensus        --CCCCCCCCAGCCCAATGTGAAATGAGCGAGTGGGGGCCGTGGGGCCCTTGCTCCAGAAGGAGAAAGCTGTGCGGCTTCCGGAAGGGCAACAAGGAGCGTTCCCGGTGGATCCTGCAGGTGATTT

                 823       833       843       853       863       873       883       893       903       913       923             941
1. Pvi RSPO1 Ref --........................................................................................................................C.
                                                                                                                        Primer R1
2. BAC 100-O13   --.......................................................................................................
3. BAC 215-D15   --...............................................................................
4. Female 1      --............................................................................Y...............................................
5. Female 2      --............................................................................Y...............................................
6. Female 3      --.....................................................................Y.............................Y........................
7. Male 1        --............................................................................................................................
8. Male 2        --............................................................................................................................
9. Male 3        NC............................................................................C...............................................
```
